# How Does Walkability Change Behavior? A Comparison between Different Age Groups in the Netherlands

**DOI:** 10.3390/ijerph17020540

**Published:** 2020-01-15

**Authors:** Bojing Liao, Pauline E. W. van den Berg, Pieter J. V. van Wesemael, Theo A. Arentze

**Affiliations:** Department of the Built Environment, Eindhoven University of Technology, PO Box 513, 5600MB Eindhoven, The Netherlands; P.E.W.v.d.Berg@tue.nl (P.E.W.v.d.B.); p.j.v.v.wesemael@tue.nl (P.J.V.v.W.); T.A.Arentze@tue.nl (T.A.A.)

**Keywords:** walkability, age groups, path analysis, trip generation, destination choice, mode choice and walking trips

## Abstract

Empirical research provides evidence that, in neighborhoods with higher walkability, individuals make more walking trips. However, it is not clear what the exact nature is of the relationships between neighborhood walkability and walking trips, since a higher walking frequency can be explained in different ways. This study examined whether the extra walking trips in better walkable neighborhoods are related primarily to trip generation, destination choice, or transport mode choice and whether this is the same for different age groups. A neighborhood fixed effects regression analysis was conducted in a first step to obtain a walkability measure for each neighborhood in the Netherlands including systematic as well as unobserved effects. Subsequently, the estimated fixed effects were used as walkability data for a path analysis based on a causal model to test the hypotheses stated. The results of the path analysis show direct relationships of neighborhood walkability with trip generation, destination choice, and transport mode choice, after controlling for the mutual relationships between the activity and trip variables. Comparing different age groups (i.e., children, adults, and elderly), the differences found mostly concerned the relationship between neighborhood walkability and trip generation. We concluded therefore that conditions for walkability are not the same for all age groups.

## 1. Introduction

Empirical research has shown that the design of the built environment has a significant influence on the extent to which individuals walk which is an important element of a healthy lifestyle [1,2,3,4]. Commonly, this influence is captured in the notion of walkability which represents the extent to which the built environment is conducive to walking. An important theoretical basis for measuring walkability is provided by Cervero et al. (2010) [5] who distinguish six built-environment dimensions, i.e., density, diversity, design, distance, destination, and demand management. Using this framework, indices of walkability have been developed to predict levels of inhabitants’ walking activity and active travel in the neighborhood [6,7,8]. These existing measures consider neighborhood features, such as dwelling density, presence of local amenities (schools, shops, etc.), and street connectivity, as determining factors of walkability. These neighborhood features correlate with the extent to which individuals are inclined to walk in their direct environment for transport or recreational purposes [1,2,3,4]. Neighborhood studies have shown that, in neighborhoods with higher walkability, individuals make more walking trips [7,9,10,11].

However, it is not clear what the exact nature is of the relationship between neighborhood walkability and walking trips, since logically a higher walking frequency can be triggered in different ways: (1) choice of activity—individuals conduct more outdoor activities and, therefore, make more walking trips; (2) choice of destination—individuals choose more often short distance destinations for their trips and, consequently, walk more; (3) choice of transport mode—individuals choose walking more often as transport mode. In other words, walkability could lead to more walking trips through an activity choice, a destination choice, or a mode choice effect.

Previous studies emphasized the relationships between walkability, outdoor activities, and short distance destinations [12,13,14,15]. However, little literature has focused on the question of how the relationship between walkability and frequency of walking trips is caused. Hence, it is not clear whether high walkability neighborhoods stimulate primarily outdoor activities, short-distance destinations, walking mode, or combinations of those. Moreover, there is empirical evidence that the association between walking and walkability may be different among age groups. Especially, children and the elderly may respond differently to features of the built environment in that respect [16,17,18,19,20]. For example, children might not have a high walking frequency in neighborhoods that score high on walkability indices, as those neighborhoods are characterized by high building and population densities that offer few places, such as green yards, where children prefer to play [20]. For the elderly, walkability has been found to be positively associated with walking for transportation and unrelated to outdoor activities [12]. Therefore, it is important to acknowledge that the relationships between walkability, outdoor activities, short-distance destinations, and walking mode may not be the same for different age groups. The purpose of the present study is to analyze these relationships to obtain a better understanding of how walkability influences activity–travel behavior of different age groups (i.e., children, adults, and elderly). This study used data from the Netherlands [21] and path analysis to answer this research question.

The remainder of the paper is organized as follows. In the next section, the existing literature is reviewed and the hypotheses and causal model that is the basis for a path analysis are outlined. Section 3 describes the data used for path analysis. The modeling results are presented in Section 4. The final section summarizes the results and discusses the implications of the findings for urban planning and neighborhood design.

## 2. Literature Review and Hypotheses

### 2.1. The Influence of Walkability on Walking Behavior and Activity

Empirical studies have examined the relationship between walkability and walking behavior, and found different correlates between built environment factors and walking for transport, recreation and exercise [22,23,24]. From the perspective of urban planning and transportation research, ease of pedestrian access to nearby destinations is related to walking behavior [1,22,23,24]. In public health literature, studies showed that some components of neighborhood walkability (e.g., access to recreation facilities and aesthetics) are related to recreational physical activity and walking [2,22,25]. Correlations are found between walkability, destination choice, and mode choice. For example, residents who live in more walkable neighborhoods tend to make more frequent walking trips to nearby destinations (e.g., the neighborhood grocery store) [22].

However, less research has examined the association between walkability and destination choice of pedestrians and whether, indeed, walkability leads to higher walking frequencies through enabling people to choose more often short-distance destinations for their trips. Some studies indicated that, with more short-distance destinations available, people would walk more often [26,27]. Similarly, Cao et al. (2006) [28] found that, in neighborhoods with stores within walking distance, residents’ frequency of walking to the stores was larger. Furthermore, Sugiyama et al. (2010) [29] found that short distance to some destinations (e.g., attractive open spaces) was associated with more recreational walking. For example, a high-quality park within walking distance of one’s home promotes walking for recreation [30]. Likewise, Chikaraishi et al. (2015) found that residents were more likely to choose walking for short-distance trips in a high walkability neighborhood [31,32]. Although these studies established the existence of associations between walkability and destination choice, other components were left out of consideration. In addition, neighborhood walkability is also related to residents’ outdoor activities. Several studies found that walkability is one of the environmental factors exerting a strong influence on out-of-home activity levels [33,34,35,36].

In addition, a number of studies indicated that the association between walkability and outdoor activities differ among different age groups. Particularly, children and the elderly display specific behavior [16,17,18,19,20]. These studies used walkability variables (e.g., residential density, land-use mix, and intersection density) to evaluate walkability and to explain further how the neighborhood environment affects children and older adults’ walking frequency [32,34,35]. For instance, positive relations have been reported between neighborhood walkability and older adults’ transport-related walking while no clear relations between neighborhood walkability and children’s walking activities were found in Belgium [19]. Furthermore, several studies have also considered the relationship between some social characteristics and out-of-home activities, and the role of neighborhood walkability [36,37]. People with low socioeconomic status are more likely to have less outdoor activities than their higher status counterparts [37].

This brief review of the existing literature suggests that neighborhood walkability plays an important role in trip generation, destination choice, and transport mode choice. Although the links among them have been discussed, it is still not clear to what extent high walkability neighborhoods stimulate trip generation, destination choice, transport mode choice, or combinations of those. It is also important to clarify whether the relationships among them are the same in different age groups. The goal of the present paper is therefore to analyze the relationships between neighborhood walkability, trip generation, destination choice, and transport mode choice in different age groups in an integrated fashion.

### 2.2. Hypotheses and Causal Model

As the brief review above indicates, the different components—activity, destination, and mode choice—have all received attention as elements of behavior that are possibly influenced by walkability. However, a systematic analysis of the relationship between walkability and walking tendency in terms of the question of whether it is primarily mediated by activity, destination or mode choice is lacking. The purpose of the present study is to provide this analysis so as to increase our understanding of the relationships between walkability and behavior.

**Figure 1:**
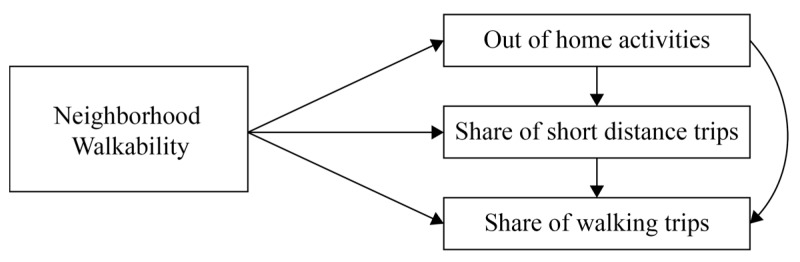
A causal model of neighborhood walkability and activity choice, destination choice, and mode choice.

Figure 1 shows the causal model that we use in a path analysis to test the possible relationships. In the model, neighborhood walkability has direct associations with out-of-home activities, the share of walking trips, and the share of short-distance trips as well as indirect associations. The indirect relationships run through the relationships between out-of-home activities and the share of short-distance trips and between the share of short-distance trips and share of walking trips. These latter vertical relationships in the figure represent well-known relationships between activity generation, trip distance, and mode choice. We are especially interested in the direct relationships between walkability and the behavioral measured variables. The hypotheses we test can be formulated as follows:

**Hypothesis** **1 (H1).** *Neighborhood walkability has a direct relationship with the number of out-of-home activities, i.e., the higher the walkability the higher the number of out-of-home activities (an activity choice effect).*

**Hypothesis** **2 (H2).** *Neighborhood walkability has a direct relationship with the share of short-distance trips, i.e., the higher the walkability the higher the share of short-distance trips after controlling for the number of out-of-home trips (a destination choice effect).*

**Hypothesis** **3 (H3).** *Neighborhood walkability has a direct relationship with the share of walking trips, i.e., the higher the walkability the higher the share of walking trips after controlling for the number of out-of-home activities and the share of short-distance trips (a mode choice effect).*

**Hypothesis** **4 (H4).** *The relationships between neighborhood walkability, out-of-home activities, the share of short-distance trips, and the share of walking trips differ among age groups.*

The findings of this study will provide a better understanding of the relationships between neighborhood walkability, out-of-home activities, short-distance destinations, and walking trips. Since we use cross-sectional data, our analysis does not allow us to identify causality.

## 3. Methods and Materials

In this section, we introduce the approaches used to measure neighborhood walkability and to analyze the relationships between neighborhood walkability, trip generation, destination choice, and transport mode choice for different age groups. For the analysis, we used data from the national travel survey in The Netherlands.

### 3.1. Approach

The analysis of the relationships between neighborhood walkability, out-of-home activities, short-distance trips, and walking trips, requires data on each of these components on the level of neighborhoods. Firstly, data on neighborhood walkability should be obtained. An obvious way to obtain walkability data would be to calculate walkability scores by using some existing walkability index, such as the well-known Walkability Index [38]. This index uses land use mix, residential density, retail floor area ratio, and intersection density to derive a measure walkability. However, this would not be the best way, as the scores calculated based on a walkability index do not include measurement error due to the fact of unobserved neighborhood attributes. To obtain a more complete measure taking into account observed as well as unobserved attributes, we proposed to use a separate regression analysis to derive walkability values of neighborhoods as follows.

**Figure 2:**
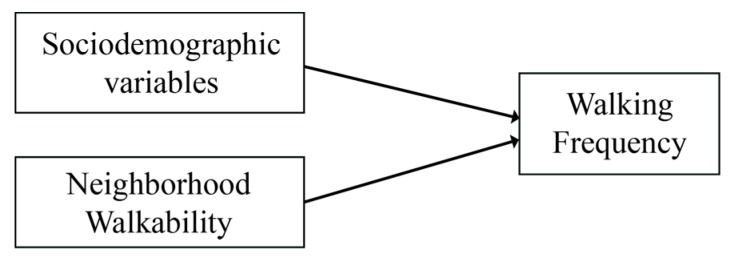
A conceptual model of the relationship between walkability variables, sociodemographic characteristics, and walking frequency [39].

The regression analysis to derive neighborhood walkability values uses individuals’ observed walking frequency as dependent variable and socio-demographic characteristics and neighborhood dummy variables (fixed effects) as independent variables. Figure 2 shows a conceptual model that represents the relationships between walkability, socio-demographic characteristics, and walking frequency [39]. As implied by this model, the total influence of walkability variables can be identified in this regression analysis as the fixed effects of neighborhoods on walking frequency after controlling for socio-demographic characteristics of individuals. The socio-demographic variables available in the survey and included in the model include gender, age, income, household composition, education, and migration background. When controlled for these socio-demographic variables, the fixed neighborhood effects in the regression model should represent the component of physical characteristics which we identify as the degree of the walkability of each neighborhood. Note that individuals are the unit of analysis in this regression analysis, to eventually obtain neighborhood-level values (the fixed effects).

In statistics, a fixed effects model refers to a regression model in which the group means are fixed (non-random) by the sample from a population [40]. The specification of the fixed-effects model, in this case, can be written as follows:

(1) $$Y_{ij}=X_{ij}\beta \;+\;\alpha_{j}\;+\;\mu_{ij\;}\;\mathrm{for}\;j=1,\ldots \ldots ,J\;and\;i=1,\ldots \ldots ,\;N$$

where $Y_{ij}$ is the observed frequency of walking trips of an individual *i* at neighborhood *j*, $X_{ij}$ is a vector of socio-demographic characteristics of that individual in that neighborhood and β is the related vector of coefficients, and $\alpha_{j}$ is an unobserved neighborhood effect and $\mu_{it}$ is an error term.

For the estimated values of the fixed effects, α represents the neighborhood walkability values. They will be used as walkability data for the path analysis based on the causal model (Figure 1) to test the hypotheses stated. Several limitations of this way of measurement should be mentioned. First, it should be noted that the fixed effects may also capture the collective preferences of individuals living in the same neighborhood. In this case, collective preferences may arise due to a self-selection effect where people who prefer walking choose to live in neighborhoods that have high walkability. Thus, a common attitude will be captured by the fixed-effect term as well making that a causal interpretation of the relationship between walkability and walking frequency is problematic. This is an inherent limitation of the cross-sectional data analysis. Furthermore, it is noted that the accuracy of this measurement method depends on the extent to which the selection of socio-demographic variables, $X_{ij}$, covers the neighborhood population characteristics that have an influence on activity and travel preferences. When important socio-demographics are missing the fixed-effect term may also capture differences in walking frequency that are related to differences in population composition of neighborhoods rather than just walkability. Therefore, we included a complete set of socio-demographic variables that are known to be influential and that (for that reason) are available generally in travel surveys.

### 3.2. Data and Variables

For the regression analysis and path analysis, we use national travel survey data from The Netherlands (OViN) [21]. This travel survey provides for a large nation-wide sample trip-diary data for a random day where all days of the week are covered. The neighborhood of each individual can be identified based on the postcode of the home address, which is available in the survey data on a 4 digit level. To increase the number of observations, four years (2013–2016) of this survey were merged for the present analysis. In the Netherlands, as in other developed countries, possible changes in physical neighborhood characteristics, such as infrastructure, land-use and accessibility to facilities, which have an influence on walkability, in a period of four years will be only minor. So, it was expected that the error caused by not taken possible changes into account will be neglectable. Hence, the data of 125,934 individuals in total were used for estimating the fixed effects model as well as the path model. As said, the socio-demographic variables used in the analysis included age, gender, income, household situation, employment situation, and migration background. The description of the variables and descriptive statistics are shown in Table 1 and Table 2.

The second step is to estimate the causal model and test the stated hypotheses by path analysis. Path analysis is a special case of structural equation modeling (SEM) that does not deal with latent variables but only with measured variables [41]. Using the same travel survey data, the number of out-of-home activities, the share of short-distance trips and the share of walking trips can be derived from the trip data for each individual on the observed day. To determine the share of short-distance trips, we used 1000 m as a cut-off-point. Thus, the ratio of the number of trips shorter than 1000 m and the total number of trips from home was calculated for each person in the database. Next, the data of the travel mode were used to calculate the share of trips made on foot. This yielded data on out-of-home activities, short distance trips, and walking trips on an individual level. However, the unit of analysis of the path analysis was the neighborhood. By computing averages of the variables (number of out-of-home activities, the share of short-distance trips, and share of walking trips) across individuals within (4-digit) postcode areas, the relevant trip data were aggregated to the postcode area level. To test hypothesis H1–H4, a path analysis was conducted for each of the three age groups (children group, adult group, and elderly group) separately, as well as all age groups together. The children group included respondents who were aged under 18, the adult group included respondents who were aged between 19 and 65, and the elderly group included respondents who are aged 65 or older. To obtain neighborhood data, the aggregation from individuals to neighborhoods (postcode areas) was conducted for each of the three age groups separately as well as all age groups together. The description and descriptive statistics of the variables are shown in Table 3 and Table 4.

### 3.3. Data Analysis

With the walkability fixed score (estimated by the fixed effects model) and measured variables, the path analysis was carried out to estimate the relationships in the causal model (Figure 1). Firstly, we analyzed these relationships across all age groups. Next, we repeated the analysis separately for each of the three age groups distinguished. The Netherlands counts in total 3829 postcode areas. However, not for all postcode areas sufficient observations (from the four years survey data) were obtained. In order to obtain meaningful averages for the variables, a minimum number of observations (individuals) was set for a postcode area to be included in the analysis. The choice of the minimum was set to 15 persons per postcode in the age group(s) considered. To determine the robustness of the model for variation in the setting, the path analysis was repeated for both a lower minimum of 10 persons and a higher minimum of 20 persons. Although the analysis results showed some differences in estimated values between the settings, the relations between walkability fixed score and measured variables are approximately the same. In other words, the results are robust for the choice of the minimum. Using this cut-off-point (number of persons ≥ 15) data for a total of 2354 postcode areas for all age groups, 800 postcode areas for the children group, 1662 postcode areas for the adult group, and 677 postcode areas for the elderly group could be used in the path analysis.

## 4. Results and Discussion

**Figure 3:**
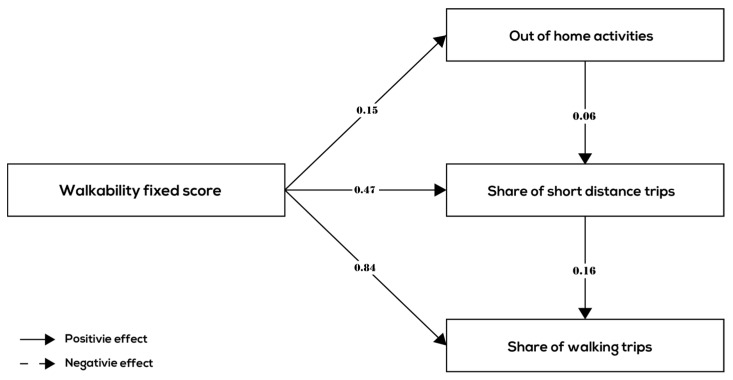
Significant relationships between walkability and behavior variables (all age groups).

**Figure 4:**
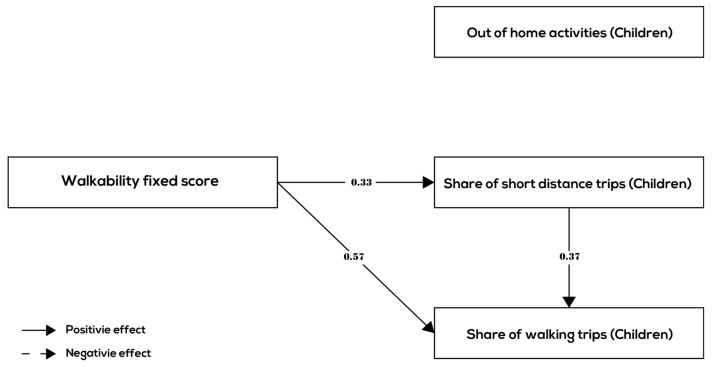
Significant relationships between walkability and behavior variables (children group).

**Figure 5:**
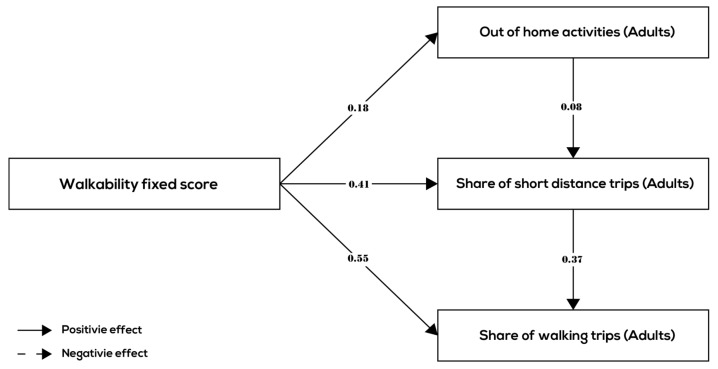
Significant relationships between walkability and behavior variables (adult group).

This section presents the results of the path analysis. The results of the path analysis are shown in Figure 3, Figure 4 and Figure 5 and Table 5 for all age groups together and the three age groups (children, adults, and elderly) separately, respectively.

### 4.1. The Relationship between Neighborhood Walkability and Behavior Variables—All Age Groups

Regarding the relationship between neighborhood walkability and behavior variables of all age groups together, the results of the path analysis (Figure 3 and Table 5—All age groups) show a positive relationship between walkability and the number of out-of-home activities that people conduct, a positive and relatively strong relationship between walkability and share of short-distance trips (keeping number of activities constant) and a positive and relatively strong relationship between walkability and the share of walking trips (keeping the number of out-of-home activities and share of short-distance trips constant). These results provide support for hypotheses H1, H2, and H3, i.e., that the relationship between walkability and walking frequency originates from an activity choice effect (people choose more out-of-home activities), a destination choice effect (people choose shorter distance destinations more often), and a transport mode effect (people choose to walk more often also for farther away destinations).

Looking at the size of the path coefficients, the transport mode effect appears to be much stronger than the activity choice effect and destination choice effect (0.84 versus 0.15 and 0.47). The internal relationships between the activity–trip variables are as expected: the number of out-of-home activities had a positive association with short distance trips (0.06), and, in turn, short distance trips had a positive association with the share of walking trips (0.16). Due to the latter relationship, the relationship between walkability and the frequency of walking trips was strengthened by an indirect relationship that ran through destination choice.

### 4.2. Differences among Age Groups

**Figure 6:**
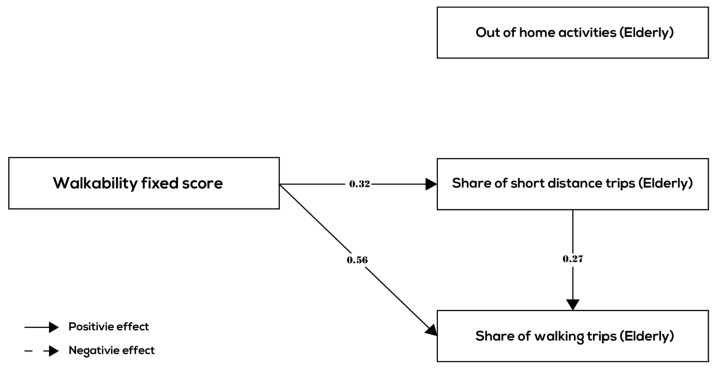
Significant relationships between walkability and behavior variables (elderly group).

Comparing the estimated path models among the different age groups (Figure 4, Figure 5 and Figure 6 and Table 5), we see that the relationships between walkability and behavior variables are quite different for children, adult, and the elderly. In all three groups, the relationships between walkability, the share of short-distance trips, and the share of walking trips were positive and significant and roughly of the same order of magnitude (although the mode choice effect was smaller for children and elderly compared to adults). In terms of the relationship between walkability and number of out-of-home activities, there is however a difference. In the children group and the elderly group, there are no links between walkability and number of out-of-home activities indicating that higher walkability is does not lead to more out-of-home activities for children and the elderly. In the adult group, there is a small positive association (0.18) between walkability and number of out-of-home activities. In conclusion, hypothesis H4 is partially confirmed by the results—there are differences between age groups in terms of behavior related to walkability, although all age groups make more short-distance trips and (controlling for distance) choose walking mode more often in higher walkability neighborhoods.

The line of argumentation is similar in terms of the internal relationships between the behavior variables: all age groups show a relatively strong positive (direct) association between the share of short-distance trips and share of walking trips. This means that for all age groups, the relationship between walkability and walking frequency is enhanced by an indirect relationship that runs through destination choice. The relationship between the number of out-of-home activities and the share of short-distance trips is small (adults and elderly) or absent (children). Furthermore, there is no (direct) association between the number of out-of-home activities and the choice of walking mode in any one of the age groups.

### 4.3. Discussion of Results

The absence of a relationship between walkability and out-of-home activities in the children group and the elderly group is the most striking finding. A possible explanation for this finding may be different for children and elderly. For children, it could be related to outdoor play where children prefer to have outdoor activities in spacious and vegetated yards rather than in places with high building density and high population density, which are typical for high walkability. For elderly, it may be related to parks where the elderly prefers to have outdoor activities. Those places are also not typical for high walkability [42]. If these explanations are valid, it means that what walkability is to children and elderly is not exactly the same as what walkability is to the adult group. For children and elderly, the conditions that contribute to walkability may differ when it comes to favoring outdoor activities. This does not hold for destination choice and transport mode choice—the conditions that support short-distance destinations and the choice of walking mode work out for children and elderly in the same way as for the adult group. It is generally held that highly walkable neighborhoods invite people to make walking trips just for recreation. Our analysis shows that people in high walkable neighborhoods do not or only modestly, dependent on age group, perform more activities outdoor. A much stronger relationship is that, in high walkable neighborhoods, people more often choose short-distance destinations and walking for their activities. In terms of the strength of the relationships, the mode choice effect appears to be somewhat larger than the destination choice effect.

## 5. Conclusions

This study aimed to increase our understanding of the relationships between neighborhood walkability, out-of-home activities (activity choice), short-distance trips (destination choice), and walking trips (mode choice). Based on data from a large national travel survey on socio–demography and trips, walkability scores were derived, and a path analysis was conducted to estimate the relationships between these variables in a causal model using the neighborhood as the unit of analysis. Since behavior may differ between age groups, the path analysis was conducted for different age groups separately.

The findings indicate that there are positive relationships between neighborhood walkability, short-distance trips, and walking trips. At the same time, neighborhood walkability has a weak association with number of out-of-home activities only in the adult group. The relationships between neighborhood walkability and number of out-of-home activities are absent in the children group and the elderly group. We conclude, therefore, that relationships between walkability, out-of-home activities, short-distance trips, and walking trips are different between age groups. People in highly walkable neighborhoods do only modestly (adults) or not at all (elderly and children) perform more outdoor activities. All age groups do, however, more often have short-distance destinations (keeping all else constant) and choose walking as transport mode (keeping all else constant). A possible explanation for the absence of an activity choice effect for children and elderly is that children and elderly may prefer specific leisure spaces (e.g., spacious playground, and parks) for recreational activities which are not typically related to high walkability (e.g., shopping center or cinema). Therefore, these findings suggest that to extend the use for a wider group of people, a measure of neighborhood walkability should also consider specific demands of children and the elderly regarding walkable neighborhood design related to outdoor activities, such as green spaces and spacious playgrounds.

Although the findings shed light on the relationships between walkability and activity-travel behavior, we should also mention some limitations of this study. Firstly, since we used cross-sectional data we cannot account for possible residential self-selection. Residential self-selection means that people who prefer to walk may choose to live in a neighborhood that allows them to walk. Due to the selection effects causal interpretation of the relationships found is problematic: the causal direction is reversed when the preference to walk already existed before one lived in the neighborhood. In a recent study, Ettema and Nieuwenhuis empirically tested the relationship between people’s preference for travel mode and residential location choice, to assess the extent to which residential self-selection plays a role [43]. Based on a survey among households who had recently moved, they found only a weak relationship between travel attitude and residential location choice. They conclude that “the association between travel attitude and travel as a factor in location choice is moderate at best” [43].

This evidence suggests that, at least in the Dutch context, residential self-selection plays only a minor role as factor in explaining travel mode choice. Nevertheless, establishing the direction of causal relationships is important when the purpose is to predict the effects of interventions in the built environment for example to improve walkability. To quantify the (pure) built environment effects propensity-scoring or sample selection methods could be used, as reviewed in Mokhtarian and Van Herick [44]. Furthermore, the collection of longitudinal data that enables one to determine the time order in which changes in behavior and changes in built environment occur would offer a more fundamental basis to identify causality. The purpose of the present study was to analyze the relationships between walking behavior and walkability using path analysis. For future research it is interesting to use either one of these approaches to increase insight in the causal directions of the relationships assumed in the path model.

Secondly, the assumption that walkability can be identified as the residual term of a regression model that predicts walking frequency based on socio-demographic variables, which was used in this study, is valid to the extent that the socio-demographic variables included effectively capture differences unrelated to physical characteristics (walkability). Therefore, care was taken to include a complete set of relevant socio-demographic variables to make sure that the influence of differences in population characteristics between neighborhoods is eliminated. However, when a collective preference of individuals within a neighborhood exists that is not related to walkability variables and neither to socio-demographics then the value of the estimated fixed effect will over- or underestimate walkability. For example, a culture pro or con walking may have emerged in a neighborhood that cannot be explained by observed population variables and neither be attributed to walkability characteristics of the neighborhood. This introduces a source of measurement error—in some cases overestimating walkability and other cases underestimating it. For future research it is worthwhile to collect in addition to socio-demographic data also data on attitudes on walking preferences. Attitudes will be correlated to socio-demographics as well as to physical (walkability) characteristics, but a remaining part of variation will exist that make correction for unobserved variables possible and increase the power of the analysis. The attitudinal data will at the same time provide a means to account for residential self-selection in the analysis [44].

Thirdly, we do not see an association or only a small association between walkability and outdoor activities. Hence, it is interesting to further investigate the relationship between walkability and outdoor activities in the future. Addressing these issues will increase our understanding of walkability and ways to design neighborhoods and plan daily urban systems to stimulate walking.

## Figures and Tables

**Table 1:** Description of walking frequency and socio-demographic characteristics.

Variables	Description of Variables
Walking frequency	The total number of walking trips (in 1 day) per person;
Gender	A binary variable: male and female;
Age	Three categories for individuals: age under 18, age 19 to 65, and age older than 65;
Income situation	Three categories for individuals: households with a low income (less than € 20,000), households with a medium-income (€ 20,000–€ 50,000), and households with a high income (more than € 50,000);
Households situation	Three categories for individuals: single persons, couples without children, and couples with children;
Employed situation	A binary category variable: employed and unemployed;
Migration background	Three categories for individuals: people are indigenous, people with a western migration background, and with a non-western migration background.

**Table 2:** Descriptive analysis of dependent variable and socio-demographic variables.

Variables	Mean	SD	Min	Max	Unit
Walking frequency	0.58	1.14	0.00	13.00	times
Men	0.49	0.50	0.00	1.00	0/1
Women	0.51	0.50	0.00	1.00	0/1
Persons age under 18	0.26	0.44	0.00	1.00	0/1
Persons age between 19 to 65	0.50	0.50	0.00	1.00	0/1
Persons age older than 65	0.23	0.42	0.00	1.00	0/1
Households has a low income	0.12	0.32	0.00	1.00	0/1
Households has a medium income	0.57	0.50	0.00	1.00	0/1
Households has a high income	0.43	0.50	0.00	1.00	0/1
Employed persons	0.46	0.50	0.00	1.00	0/1
Unemployed persons	0.54	0.50	0.00	1.00	0/1
Single persons	0.14	0.34	0.00	1.00	0/1
Couples without children	0.28	0.45	0.00	1.00	0/1
Couples with children	0.58	0.49	0.00	1.00	0/1
Indigenous	0.84	0.37	0.00	1.00	0/1
Western migration background	0.07	0.27	0.00	1.00	0/1
Non-Western migration background	0.08	0.27	0.00	1.00	0/1
	*N* = 125,934 persons				

**Table 3:** Description of measured variables.

Variables	Description of Variables
Out-of-home activities	The average number of out-of-home activities of respondents in each postcode;
Share of short-distance trips	The average percentage of short-distance trips (less than 1000 m) of respondents in each postcode;
Share of walking trips	The average percentage of walking trips of respondents in each postcode.

**Table 4:** Descriptive analysis of measured variables in different age groups.

Variables	Mean	SD	Min	Max	Unit
***All age groups***					
Walkability fixed score	0.06	0.07	−0.11	0.46	-
Out-of-home activities	3.50	0.43	0.00	5.32	times
Share of short distance trips	14.91	5.90	0.00	43.86	%
Share of walking trips	15.98	6.40	0.00	58.76	%
	*N* = 2354 postcode areas (Persons ≥ 15)				
***Children group***					
Walkability fixed score	0.06	0.06	−0.06	0.46	-
Out-of-home activities (Children)	3.49	0.50	0.00	5.18	times
Share of short distance trips (Children)	21.69	8.86	0.00	58.49	%
Share of walking trips (Children)	18.71	9.09	0.00	55.56	%
	*N* = 800 postcode areas (Persons ≥ 15)				
***Adults group***					
Walkability fixed score	0.07	0.06	−0.09	0.35	-
Out-of-home activities (Adults)	3.68	0.55	0.00	5.71	times
Share of short distance trips (Adults)	11.65	5.91	0.00	33.98	%
Share of walking trips (Adults)	13.55	6.32	0.00	41.13	%
	*N* = 1662 postcode areas (Persons ≥ 15)				
***Elderly group***					
Walkability fixed score	0.06	0.05	−0.05	0.29	-
Out-of-home activities (Elderly)	3.23	0.45	1.35	4.76	times
Share of short distance trips (Elderly)	14.22	7.77	0.00	53.49	%
Share of walking trips (Elderly)	19.67	8.64	0.00	51.79	%
	*N* = 677 postcode areas (Persons ≥ 15)				

**Table 5:** Path analysis model standardized estimates.

To From	Out-of-Home Activities	Share of Short-Distance Trips	Share of Walking Trips
***All age groups***			
Walkability	0.154 ***	0.467 ***	0.839 ***
Out-of-home activities		0.059 ***	−0.054
Share of short-distance trips			0.160 ***
***Children group***			
Walkability (Children)	0.031	0.334 **	0.572 ***
Out-of-home activities (Children)		0.024	−0.062
Share of short-distance trips (Children)			0.374 ***
***Adults group***			
Walkability (Adults)	0.180 ***	0.411 ***	0.551 ***
Out-of-home activities (Adults)		0.077 ***	−0.058
Share of short-distance trips (Adults)			0.370 ***
***Elderly group***			
Walkability (Elderly)	0.044	0.317 ***	0.556 ***
Out-of-home activities (Elderly)		0.005	−0.047
Share of short-distance trips Elderly)			0.274 ***

Significance codes: ‘***’ 0.001; ‘**’ 0.01.
